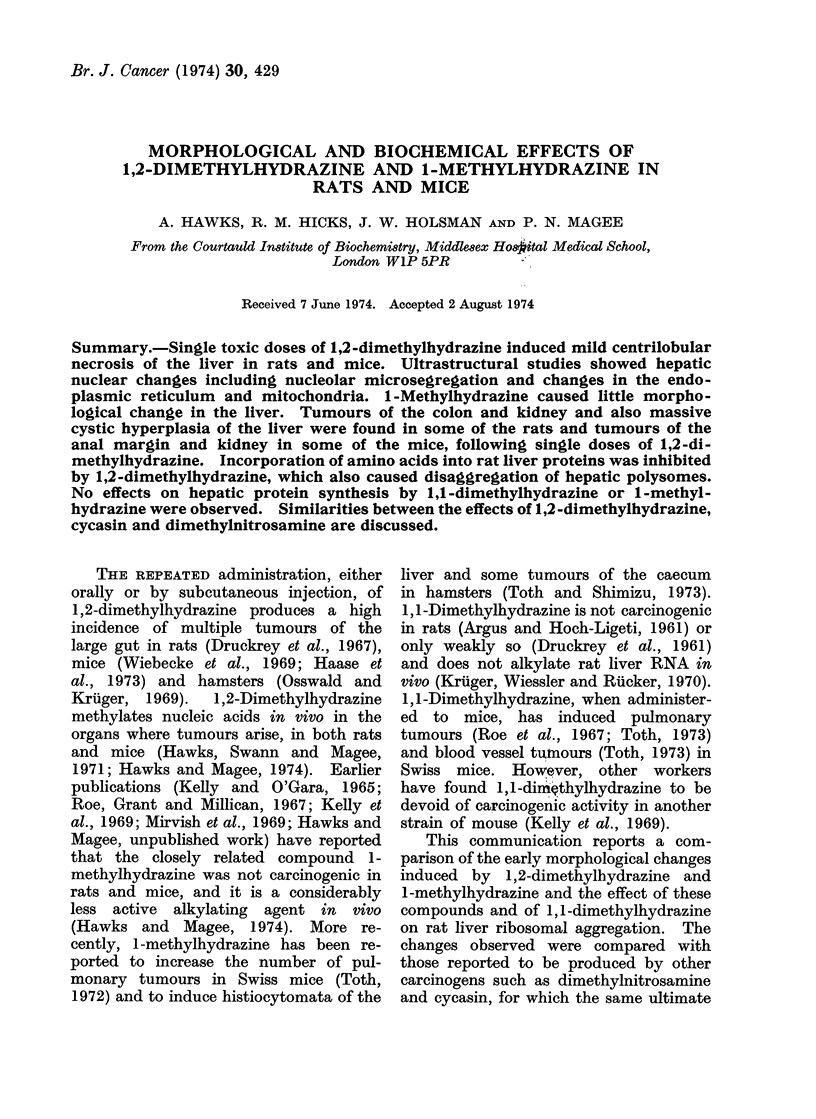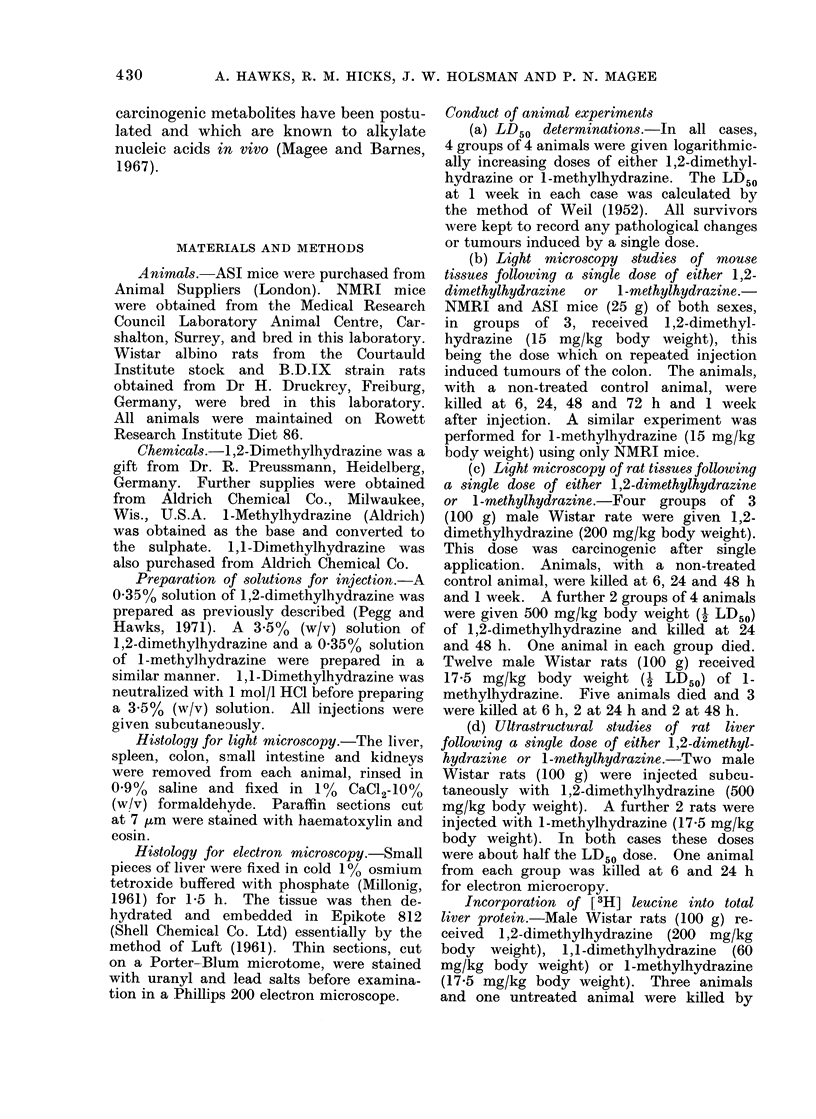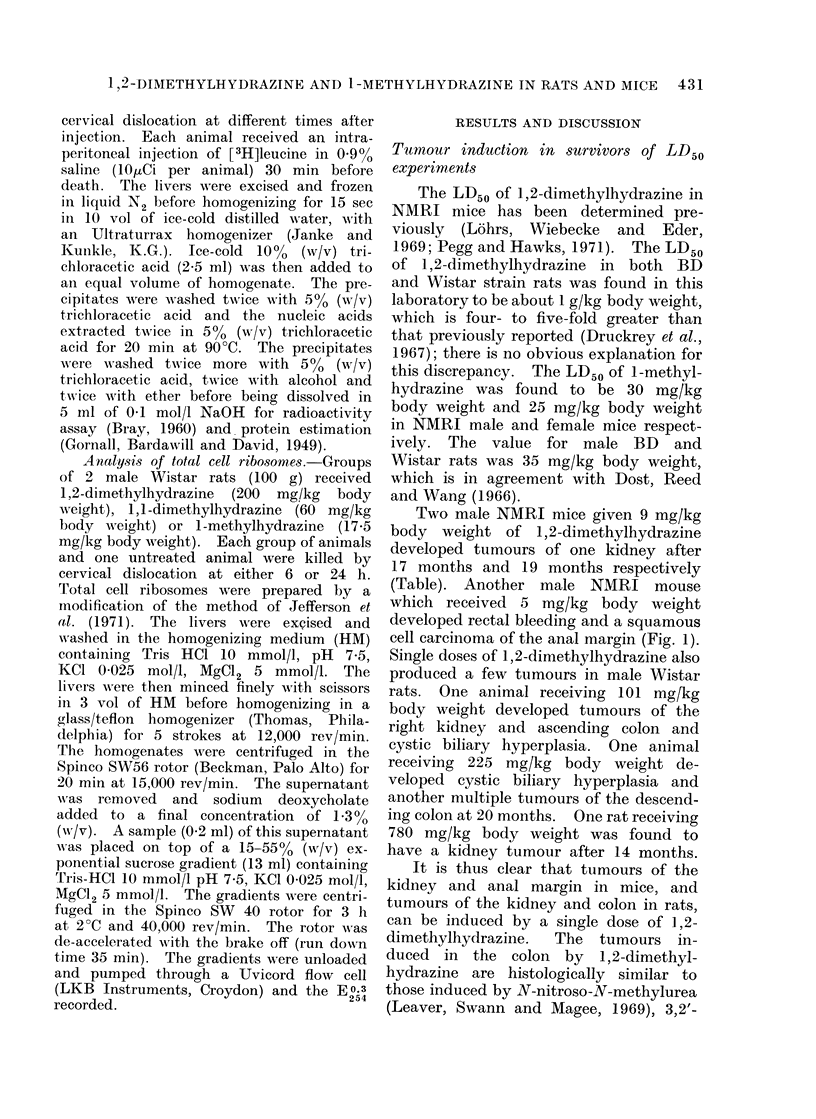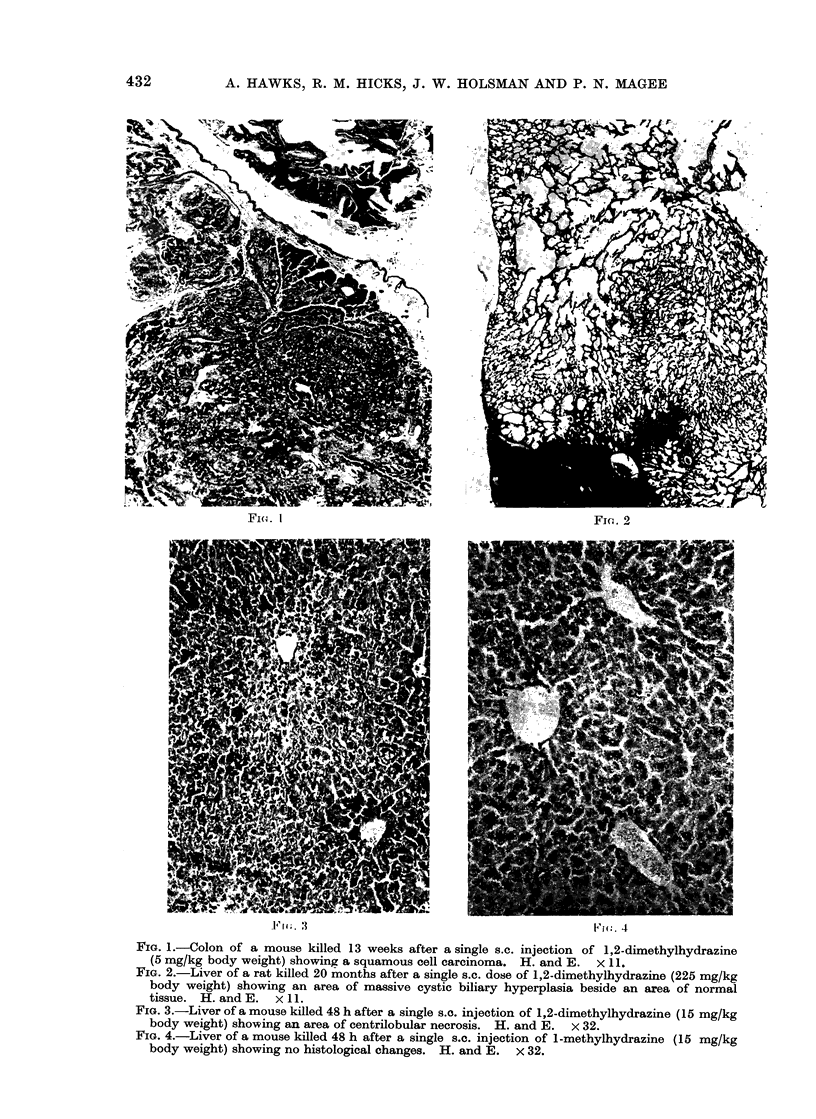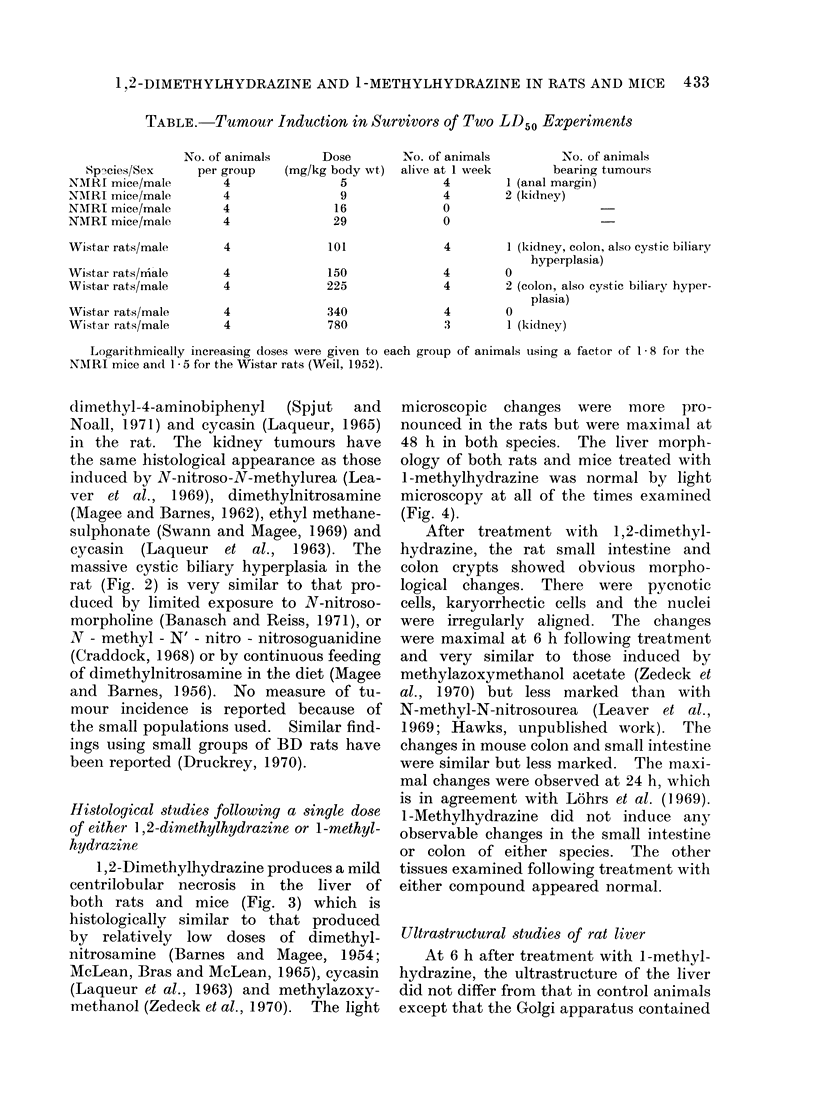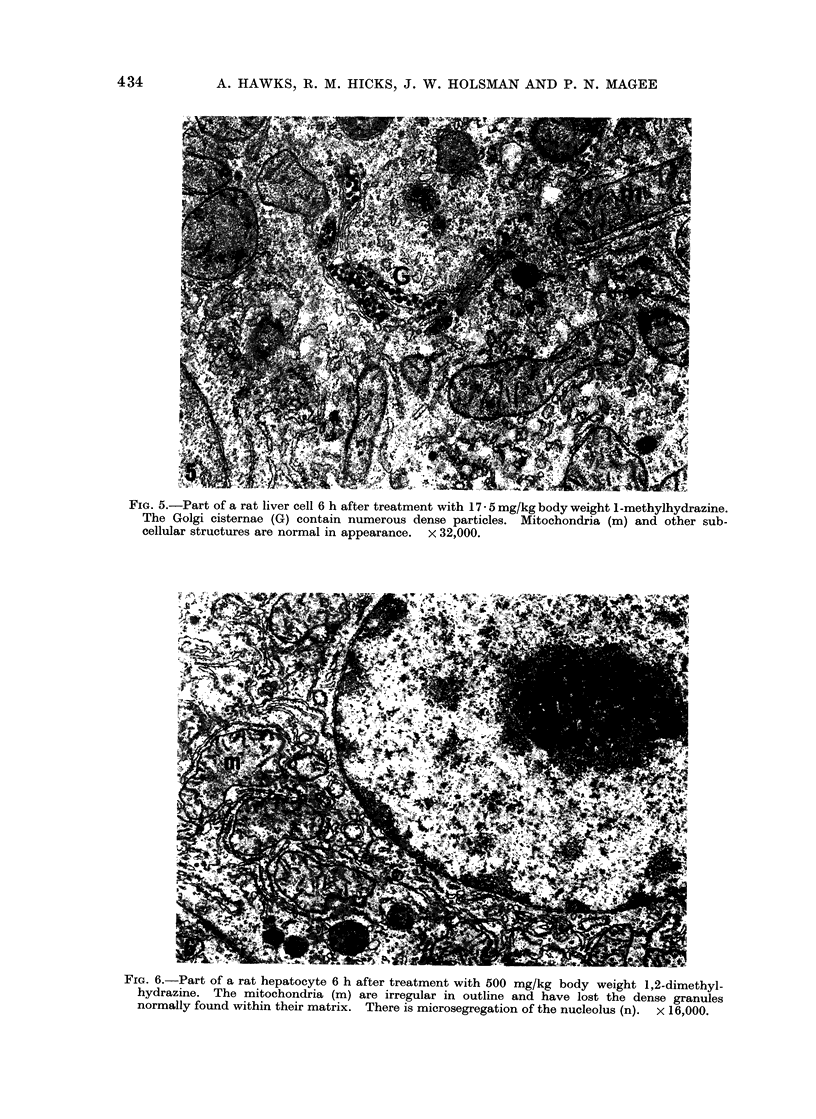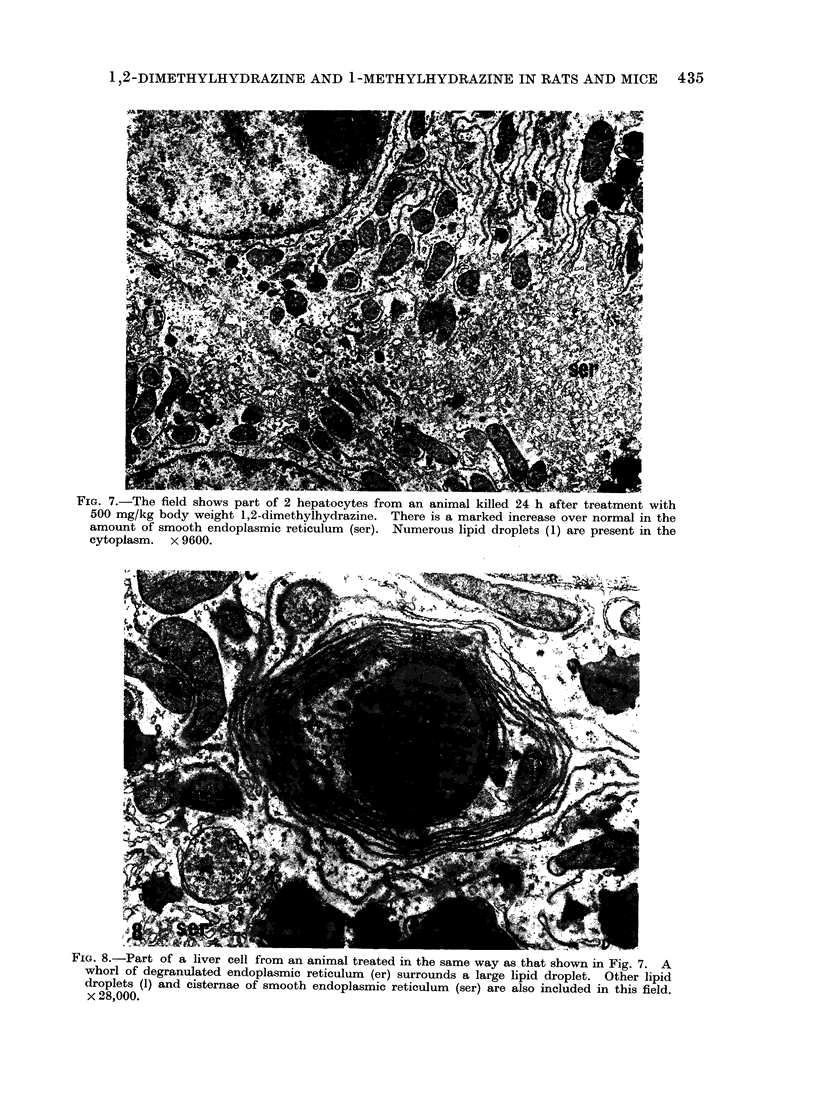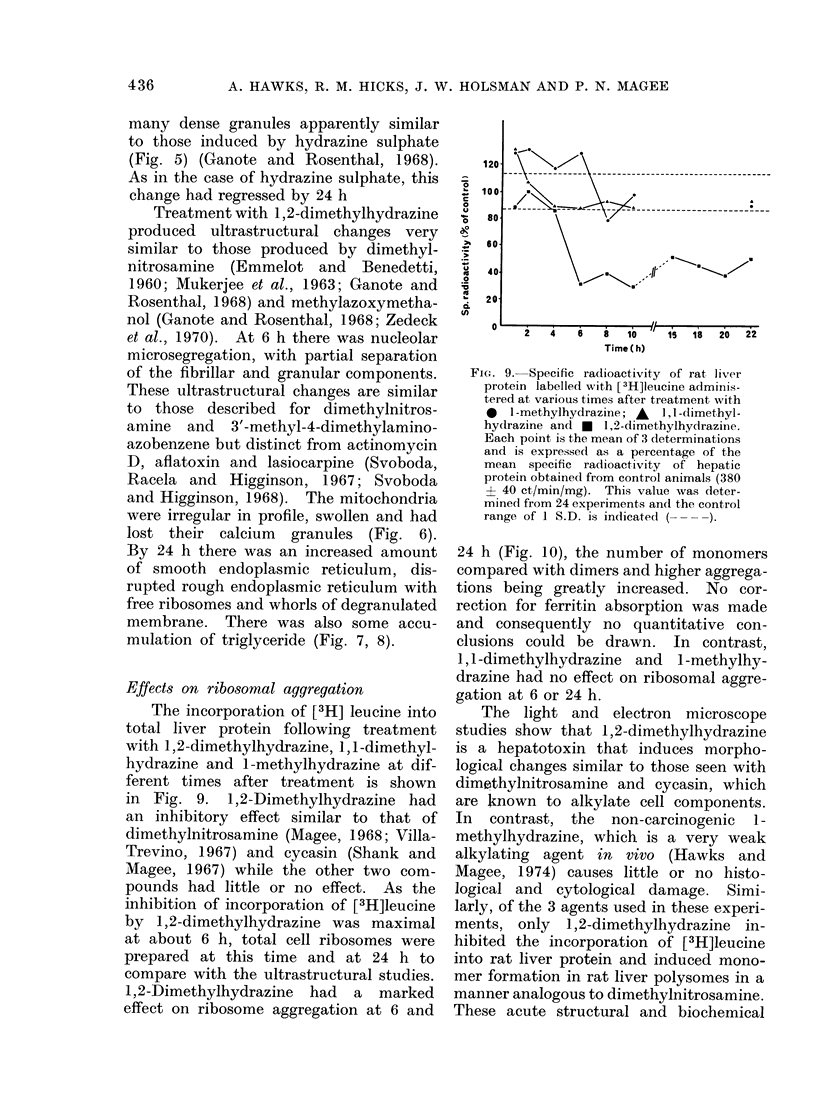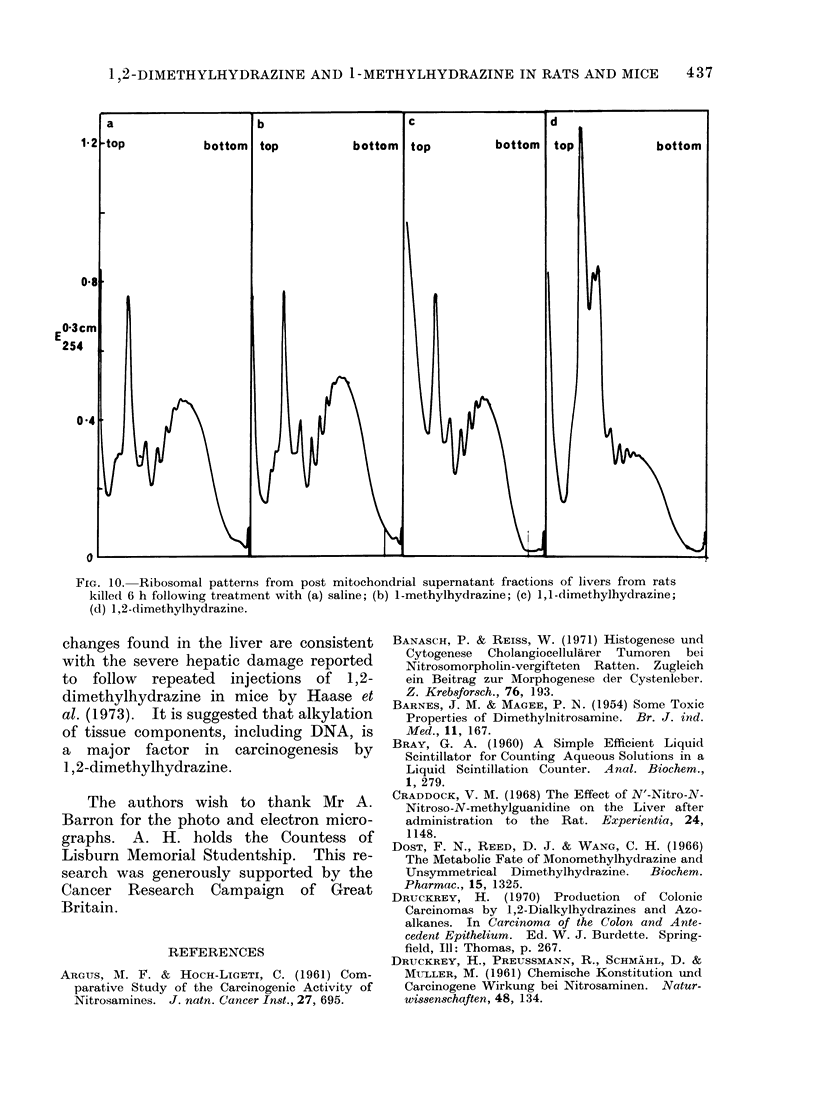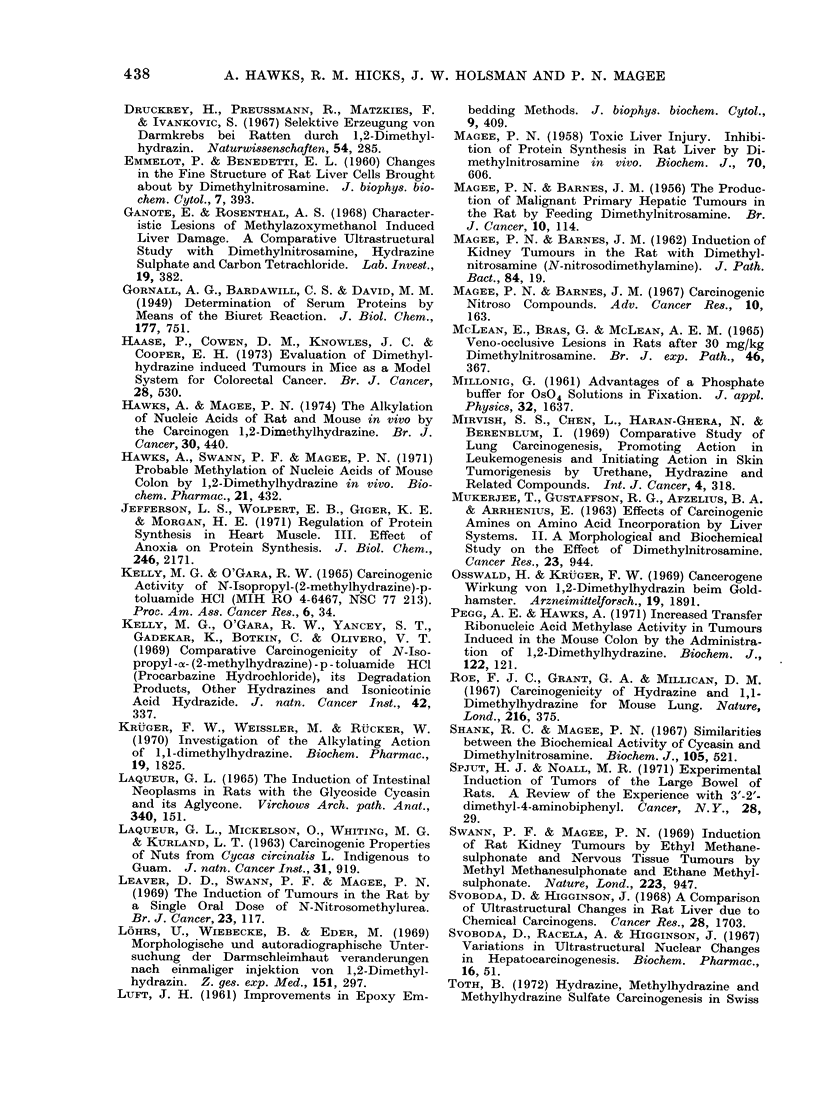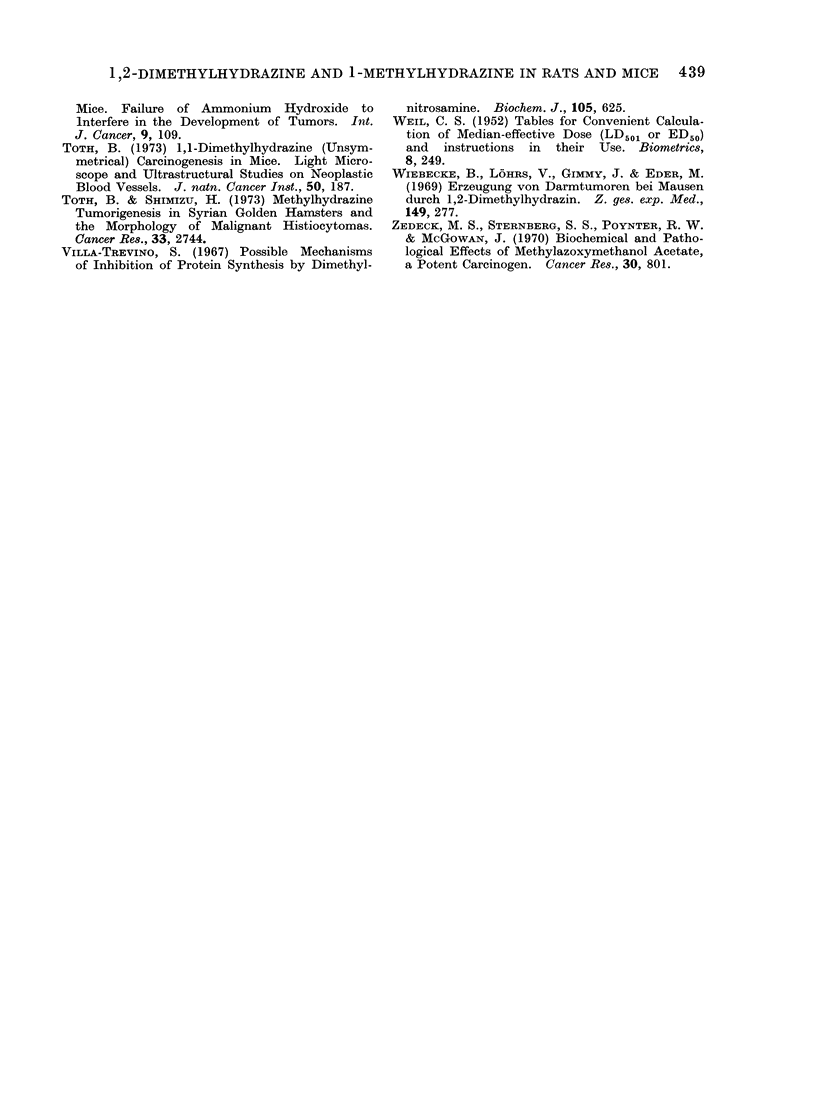# Morphological and Biochemical Effects of 1,2-Dimethylhydrazine and 1-Methylhydrazine in Rats and Mice

**DOI:** 10.1038/bjc.1974.217

**Published:** 1974-11

**Authors:** A. Hawks, R. M. Hicks, J. W. Holsman, P. N. Magee

## Abstract

**Images:**


*Br. J. Cancer* (1974) **30**, 429

### MORPHOLOGICAL AND BIOCHEMICAL EFFECTS OF 1,2-DIMETHYLHYDRAZINE AND 1-METHYLHYDRAZINE IN RATS AND MICE

A. HAWKS, R. M. HICKS, J. W. HOLSMAN AND P. N. MAGEE

*From the Courtauld Institute of Biochemistry, Middlesex Hospital Medical School, London W1P 5PR*

Received 7 June 1974. Accepted 2 August 1974

**Summary.—Single toxic doses of 1,2-dimethylhydrazine induced mild centrilobular necrosis of the liver in rats and mice. Ultrastructural studies showed hepatic nuclear changes including nucleolar microsegregation and changes in the endoplasmic reticulum and mitochondria. 1-Methylhydrazine caused little morphological change in the liver. Tumours of the colon and kidney and also massive cystic hyperplasia of the liver were found in some of the rats and tumours of the anal margin and kidney in some of the mice, following single doses of 1,2-dimethylhydrazine. Incorporation of amino acids into rat liver proteins was inhibited by 1,2-dimethylhydrazine, which also caused disaggregation of hepatic polysomes. No effects on hepatic protein synthesis by 1,1-dimethylhydrazine or 1-methylhydrazine were observed. Similarities between the effects of 1,2-dimethylhydrazine, cycasin and dimethylnitrosamine are discussed.**

THE REPEATED administration, either orally or by subcutaneous injection, of 1,2-dimethylhydrazine produces a high incidence of multiple tumours of the large gut in rats (Druckrey *et al.*, 1967), mice (Wiebecke *et al.*, 1969; Haase *et al.*, 1973) and hamsters (Osswald and Krüger, 1969). 1,2-Dimethylhydrazine methylates nucleic acids *in vivo* in the organs where tumours arise, in both rats and mice (Hawks, Swann and Magee, 1971; Hawks and Magee, 1974). Earlier publications (Kelly and O'Gara, 1965; Roe, Grant and Millican, 1967; Kelly *et al.*, 1969; Mirvish *et al.*, 1969; Hawks and Magee, unpublished work) have reported that the closely related compound 1-methylhydrazine was not carcinogenic in rats and mice, and it is a considerably less active alkylating agent *in vivo* (Hawks and Magee, 1974). More recently, 1-methylhydrazine has been reported to increase the number of pulmonary tumours in Swiss mice (Toth, 1972) and to induce histiocytomata of the liver and some tumours of the caecum in hamsters (Toth and Shimizu, 1973). 1,1-Dimethylhydrazine is not carcinogenic in rats (Argus and Hoch-Ligeti, 1961) or only weakly so (Druckrey *et al.*, 1961) and does not alkylate rat liver RNA *in vivo* (Krüger, Wiessler and Rücker, 1970). 1,1-Dimethylhydrazine, when administered to mice, has induced pulmonary tumours (Roe *et al.*, 1967; Toth, 1973) and blood vessel tumours (Toth, 1973) in Swiss mice. However, other workers have found 1,1-dimethylhydrazine to be devoid of carcinogenic activity in another strain of mouse (Kelly *et al.*, 1969).

This communication reports a comparison of the early morphological changes induced by 1,2-dimethylhydrazine and 1-methylhydrazine and the effect of these compounds and of 1,1-dimethylhydrazine on rat liver ribosomal aggregation. The changes observed were compared with those reported to be produced by other carcinogens such as dimethylnitrosamine and cycasin, for which the same ultimate carcinogenic metabolites have been postulated and which are known to alkylate nucleic acids *in vivo* (Magee and Barnes, 1967).

### MATERIALS AND METHODS

*Animals.*—ASI mice were purchased from Animal Suppliers (London). NMRI mice were obtained from the Medical Research Council Laboratory Animal Centre, Carshalton, Surrey, and bred in this laboratory. Wistar albino rats from the Courtauld Institute stock and B.D.IX strain rats obtained from Dr H. Druckrey, Freiburg, Germany, were bred in this laboratory. All animals were maintained on Rowett Research Institute Diet 86.

*Chemicals.*—1,2-Dimethylhydrazine was a gift from Dr. R. Preussmann, Heidelberg, Germany. Further supplies were obtained from Aldrich Chemical Co., Milwaukee, Wis., U.S.A. 1-Methylhydrazine (Aldrich) was obtained as the base and converted to the sulphate. 1,1-Dimethylhydrazine was also purchased from Aldrich Chemical Co.

*Preparation of solutions for injection.*—A 0·35% solution of 1,2-dimethylhydrazine was prepared as previously described (Pegg and Hawks, 1971). A 3·5% (w/v) solution of 1,2-dimethylhydrazine and a 0·35% solution of 1-methylhydrazine were prepared in a similar manner. 1,1-Dimethylhydrazine was neutralized with 1 mol/l HCl before preparing a 3·5% (w/v) solution. All injections were given subcutaneously.

*Histology for light microscopy.*—The liver, spleen, colon, small intestine and kidneys were removed from each animal, rinsed in 0·9% saline and fixed in 1% $CaCl_2$-10% (w/v) formaldehyde. Paraffin sections cut at 7 μm were stained with haematoxylin and eosin.

*Histology for electron microscopy.*—Small pieces of liver were fixed in cold 1% osmium tetroxide buffered with phosphate (Millonig, 1961) for 1·5 h. The tissue was then dehydrated and embedded in Epikote 812 (Shell Chemical Co. Ltd) essentially by the method of Luft (1961). Thin sections, cut on a Porter-Blum microtome, were stained with uranyl and lead salts before examination in a Phillips 200 electron microscope.

*Conduct of animal experiments*

(a) *$LD_{50}$ determinations.*—In all cases, 4 groups of 4 animals were given logarithmically increasing doses of either 1,2-dimethylhydrazine or 1-methylhydrazine. The $LD_{50}$ at 1 week in each case was calculated by the method of Weil (1952). All survivors were kept to record any pathological changes or tumours induced by a single dose.

(b) *Light microscopy studies of mouse tissues following a single dose of either* 1,2-*dimethylhydrazine or* 1-*methylhydrazine.*—NMRI and ASI mice (25 g) of both sexes, in groups of 3, received 1,2-dimethylhydrazine (15 mg/kg body weight), this being the dose which on repeated injection induced tumours of the colon. The animals, with a non-treated control animal, were killed at 6, 24, 48 and 72 h and 1 week after injection. A similar experiment was performed for 1-methylhydrazine (15 mg/kg body weight) using only NMRI mice.

(c) *Light microscopy of rat tissues following a single dose of either* 1,2-*dimethylhydrazine or* 1-*methylhydrazine.*—Four groups of 3 (100 g) male Wistar rate were given 1,2-dimethylhydrazine (200 mg/kg body weight). This dose was carcinogenic after single application. Animals, with a non-treated control animal, were killed at 6, 24 and 48 h and 1 week. A further 2 groups of 4 animals were given 500 mg/kg body weight ($\frac{1}{2}$ $LD_{50}$) of 1,2-dimethylhydrazine and killed at 24 and 48 h. One animal in each group died. Twelve male Wistar rats (100 g) received 17·5 mg/kg body weight ($\frac{1}{2}$ $LD_{50}$) of 1-methylhydrazine. Five animals died and 3 were killed at 6 h, 2 at 24 h and 2 at 48 h.

(d) *Ultrastructural studies of rat liver following a single dose of either* 1,2-*dimethylhydrazine or* 1-*methylhydrazine.*—Two male Wistar rats (100 g) were injected subcutaneously with 1,2-dimethylhydrazine (500 mg/kg body weight). A further 2 rats were injected with 1-methylhydrazine (17·5 mg/kg body weight). In both cases these doses were about half the $LD_{50}$ dose. One animal from each group was killed at 6 and 24 h for electron microcropy.

*Incorporation of* [$^3$H] *leucine into total liver protein.*—Male Wistar rats (100 g) received 1,2-dimethylhydrazine (200 mg/kg body weight), 1,1-dimethylhydrazine (60 mg/kg body weight) or 1-methylhydrazine (17·5 mg/kg body weight). Three animals and one untreated animal were killed by cervical dislocation at different times after injection. Each animal received an intraperitoneal injection of [$^3$H]leucine in 0·9% saline (10$\mu$Ci per animal) 30 min before death. The livers were excised and frozen in liquid $N_2$ before homogenizing for 15 sec in 10 vol of ice-cold distilled water, with an Ultraturrax homogenizer (Janke and Kunkle, K.G.). Ice-cold 10% (w/v) trichloracetic acid (2·5 ml) was then added to an equal volume of homogenate. The precipitates were washed twice with 5% (w/v) trichloracetic acid and the nucleic acids extracted twice in 5% (w/v) trichloracetic acid for 20 min at 90°C. The precipitates were washed twice more with 5% (w/v) trichloracetic acid, twice with alcohol and twice with ether before being dissolved in 5 ml of 0·1 mol/l NaOH for radioactivity assay (Bray, 1960) and protein estimation (Gornall, Bardawill and David, 1949).

*Analysis of total cell ribosomes.*—Groups of 2 male Wistar rats (100 g) received 1,2-dimethylhydrazine (200 mg/kg body weight), 1,1-dimethylhydrazine (60 mg/kg body weight) or 1-methylhydrazine (17·5 mg/kg body weight). Each group of animals and one untreated animal were killed by cervical dislocation at either 6 or 24 h. Total cell ribosomes were prepared by a modification of the method of Jefferson *et al.* (1971). The livers were excised and washed in the homogenizing medium (HM) containing Tris HCl 10 mmol/l, pH 7·5, KCl 0·025 mol/l, $MgCl_2$ 5 mmol/l. The livers were then minced finely with scissors in 3 vol of HM before homogenizing in a glass/teflon homogenizer (Thomas, Philadelphia) for 5 strokes at 12,000 rev/min. The homogenates were centrifuged in the Spinco SW56 rotor (Beckman, Palo Alto) for 20 min at 15,000 rev/min. The supernatant was removed and sodium deoxycholate added to a final concentration of 1·3% (w/v). A sample (0·2 ml) of this supernatant was placed on top of a 15–55% (w/v) exponential sucrose gradient (13 ml) containing Tris-HCl 10 mmol/l pH 7·5, KCl 0·025 mol/l, $MgCl_2$ 5 mmol/l. The gradients were centrifuged in the Spinco SW 40 rotor for 3 h at 2°C and 40,000 rev/min. The rotor was de-accelerated with the brake off (run down time 35 min). The gradients were unloaded and pumped through a Uvicord flow cell (LKB Instruments, Croydon) and the $E_{254}^{0.3}$ recorded.

### RESULTS AND DISCUSSION

### *Tumour induction in survivors of $LD_{50}$ experiments*

The $LD_{50}$ of 1,2-dimethylhydrazine in NMRI mice has been determined previously (Löhrs, Wiebecke and Eder, 1969; Pegg and Hawks, 1971). The $LD_{50}$ of 1,2-dimethylhydrazine in both BD and Wistar strain rats was found in this laboratory to be about 1 g/kg body weight, which is four- to five-fold greater than that previously reported (Druckrey *et al.*, 1967); there is no obvious explanation for this discrepancy. The $LD_{50}$ of 1-methylhydrazine was found to be 30 mg/kg body weight and 25 mg/kg body weight in NMRI male and female mice respectively. The value for male BD and Wistar rats was 35 mg/kg body weight, which is in agreement with Dost, Reed and Wang (1966).

Two male NMRI mice given 9 mg/kg body weight of 1,2-dimethylhydrazine developed tumours of one kidney after 17 months and 19 months respectively (Table). Another male NMRI mouse which received 5 mg/kg body weight developed rectal bleeding and a squamous cell carcinoma of the anal margin (Fig. 1). Single doses of 1,2-dimethylhydrazine also produced a few tumours in male Wistar rats. One animal receiving 101 mg/kg body weight developed tumours of the right kidney and ascending colon and cystic biliary hyperplasia. One animal receiving 225 mg/kg body weight developed cystic biliary hyperplasia and another multiple tumours of the descending colon at 20 months. One rat receiving 780 mg/kg body weight was found to have a kidney tumour after 14 months.

It is thus clear that tumours of the kidney and anal margin in mice, and tumours of the kidney and colon in rats, can be induced by a single dose of 1,2-dimethylhydrazine. The tumours induced in the colon by 1,2-dimethylhydrazine are histologically similar to those induced by *N*-nitroso-*N*-methylurea (Leaver, Swann and Magee, 1969), 3,2′-

FIG. 1

FIG. 2

FIG. 3

FIG. 4

FIG. 1.—Colon of a mouse killed 13 weeks after a single s.c. injection of 1,2-dimethylhydrazine (5 mg/kg body weight) showing a squamous cell carcinoma. H. and E. ×11.

FIG. 2.—Liver of a rat killed 20 months after a single s.c. dose of 1,2-dimethylhydrazine (225 mg/kg body weight) showing an area of massive cystic biliary hyperplasia beside an area of normal tissue. H. and E. ×11.

FIG. 3.—Liver of a mouse killed 48 h after a single s.c. injection of 1,2-dimethylhydrazine (15 mg/kg body weight) showing an area of centrilobular necrosis. H. and E. ×32.

FIG. 4.—Liver of a mouse killed 48 h after a single s.c. injection of 1-methylhydrazine (15 mg/kg body weight) showing no histological changes. H. and E. ×32.

TABLE.—*Tumour Induction in Survivors of Two $LD_{50}$ Experiments*

| Species/Sex | No. of animals per group | Dose (mg/kg body wt) | No. of animals alive at 1 week | No. of animals bearing tumours |
|---|---|---|---|---|
| NMRI mice/male | 4 | 5 | 4 | 1 (anal margin) |
| NMRI mice/male | 4 | 9 | 4 | 2 (kidney) |
| NMRI mice/male | 4 | 16 | 0 | — |
| NMRI mice/male | 4 | 29 | 0 | — |
| Wistar rats/male | 4 | 101 | 4 | 1 (kidney, colon, also cystic biliary hyperplasia) |
| Wistar rats/male | 4 | 150 | 4 | 0 |
| Wistar rats/male | 4 | 225 | 4 | 2 (colon, also cystic biliary hyperplasia) |
| Wistar rats/male | 4 | 340 | 4 | 0 |
| Wistar rats/male | 4 | 780 | 3 | 1 (kidney) |

Logarithmically increasing doses were given to each group of animals using a factor of 1·8 for the NMRI mice and 1·5 for the Wistar rats (Weil, 1952).

dimethyl-4-aminobiphenyl (Spjut and Noall, 1971) and cycasin (Laqueur, 1965) in the rat. The kidney tumours have the same histological appearance as those induced by *N*-nitroso-*N*-methylurea (Leaver *et al.*, 1969), dimethylnitrosamine (Magee and Barnes, 1962), ethyl methanesulphonate (Swann and Magee, 1969) and cycasin (Laqueur *et al.*, 1963). The massive cystic biliary hyperplasia in the rat (Fig. 2) is very similar to that produced by limited exposure to *N*-nitrosomorpholine (Banasch and Reiss, 1971), or *N* - methyl - N′ - nitro - nitrosoguanidine (Craddock, 1968) or by continuous feeding of dimethylnitrosamine in the diet (Magee and Barnes, 1956). No measure of tumour incidence is reported because of the small populations used. Similar findings using small groups of BD rats have been reported (Druckrey, 1970).

*Histological studies following a single dose of either* 1,2-*dimethylhydrazine or* 1-*methylhydrazine*

1,2-Dimethylhydrazine produces a mild centrilobular necrosis in the liver of both rats and mice (Fig. 3) which is histologically similar to that produced by relatively low doses of dimethylnitrosamine (Barnes and Magee, 1954; McLean, Bras and McLean, 1965), cycasin (Laqueur *et al.*, 1963) and methylazoxymethanol (Zedeck *et al.*, 1970). The light microscopic changes were more pronounced in the rats but were maximal at 48 h in both species. The liver morphology of both rats and mice treated with 1-methylhydrazine was normal by light microscopy at all of the times examined (Fig. 4).

After treatment with 1,2-dimethylhydrazine, the rat small intestine and colon crypts showed obvious morphological changes. There were pycnotic cells, karyorrhectic cells and the nuclei were irregularly aligned. The changes were maximal at 6 h following treatment and very similar to those induced by methylazoxymethanol acetate (Zedeck *et al.*, 1970) but less marked than with N-methyl-N-nitrosourea (Leaver *et al.*, 1969; Hawks, unpublished work). The changes in mouse colon and small intestine were similar but less marked. The maximal changes were observed at 24 h, which is in agreement with Löhrs *et al.* (1969). 1-Methylhydrazine did not induce any observable changes in the small intestine or colon of either species. The other tissues examined following treatment with either compound appeared normal.

*Ultrastructural studies of rat liver*

At 6 h after treatment with 1-methylhydrazine, the ultrastructure of the liver did not differ from that in control animals except that the Golgi apparatus contained

FIG. 5.—Part of a rat liver cell 6 h after treatment with 17·5 mg/kg body weight 1-methylhydrazine. The Golgi cisternae (G) contain numerous dense particles. Mitochondria (m) and other subcellular structures are normal in appearance. × 32,000.

FIG. 6.—Part of a rat hepatocyte 6 h after treatment with 500 mg/kg body weight 1,2-dimethylhydrazine. The mitochondria (m) are irregular in outline and have lost the dense granules normally found within their matrix. There is microsegregation of the nucleolus (n). × 16,000.

FIG. 7.—The field shows part of 2 hepatocytes from an animal killed 24 h after treatment with 500 mg/kg body weight 1,2-dimethylhydrazine. There is a marked increase over normal in the amount of smooth endoplasmic reticulum (ser). Numerous lipid droplets (l) are present in the cytoplasm. ×9600.

FIG. 8.—Part of a liver cell from an animal treated in the same way as that shown in Fig. 7. A whorl of degranulated endoplasmic reticulum (er) surrounds a large lipid droplet. Other lipid droplets (l) and cisternae of smooth endoplasmic reticulum (ser) are also included in this field. ×28,000.

many dense granules apparently similar to those induced by hydrazine sulphate (Fig. 5) (Ganote and Rosenthal, 1968). As in the case of hydrazine sulphate, this change had regressed by 24 h

Treatment with 1,2-dimethylhydrazine produced ultrastructural changes very similar to those produced by dimethylnitrosamine (Emmelot and Benedetti, 1960; Mukerjee *et al.*, 1963; Ganote and Rosenthal, 1968) and methylazoxymethanol (Ganote and Rosenthal, 1968; Zedeck *et al.*, 1970). At 6 h there was nucleolar microsegregation, with partial separation of the fibrillar and granular components. These ultrastructural changes are similar to those described for dimethylnitrosamine and 3′-methyl-4-dimethylaminoazobenzene but distinct from actinomycin D, aflatoxin and lasiocarpine (Svoboda, Racela and Higginson, 1967; Svoboda and Higginson, 1968). The mitochondria were irregular in profile, swollen and had lost their calcium granules (Fig. 6). By 24 h there was an increased amount of smooth endoplasmic reticulum, disrupted rough endoplasmic reticulum with free ribosomes and whorls of degranulated membrane. There was also some accumulation of triglyceride (Fig. 7, 8).

*Effects on ribosomal aggregation*

The incorporation of [$^3$H] leucine into total liver protein following treatment with 1,2-dimethylhydrazine, 1,1-dimethylhydrazine and 1-methylhydrazine at different times after treatment is shown in Fig. 9. 1,2-Dimethylhydrazine had an inhibitory effect similar to that of dimethylnitrosamine (Magee, 1968; Villa-Trevino, 1967) and cycasin (Shank and Magee, 1967) while the other two compounds had little or no effect. As the inhibition of incorporation of [$^3$H]leucine by 1,2-dimethylhydrazine was maximal at about 6 h, total cell ribosomes were prepared at this time and at 24 h to compare with the ultrastructural studies. 1,2-Dimethylhydrazine had a marked effect on ribosome aggregation at 6 and

FIG. 9.—Specific radioactivity of rat liver protein labelled with [$^3$H]leucine administered at various times after treatment with ● 1-methylhydrazine; ▲ 1,1-dimethylhydrazine and ■ 1,2-dimethylhydrazine. Each point is the mean of 3 determinations and is expressed as a percentage of the mean specific radioactivity of hepatic protein obtained from control animals (380 $\pm$ 40 ct/min/mg). This value was determined from 24 experiments and the control range of 1 S.D. is indicated (– – – –).

24 h (Fig. 10), the number of monomers compared with dimers and higher aggregations being greatly increased. No correction for ferritin absorption was made and consequently no quantitative conclusions could be drawn. In contrast, 1,1-dimethylhydrazine and 1-methylhydrazine had no effect on ribosomal aggregation at 6 or 24 h.

The light and electron microscope studies show that 1,2-dimethylhydrazine is a hepatotoxin that induces morphological changes similar to those seen with dimethylnitrosamine and cycasin, which are known to alkylate cell components. In contrast, the non-carcinogenic 1-methylhydrazine, which is a very weak alkylating agent *in vivo* (Hawks and Magee, 1974) causes little or no histological and cytological damage. Similarly, of the 3 agents used in these experiments, only 1,2-dimethylhydrazine inhibited the incorporation of [$^3$H]leucine into rat liver protein and induced monomer formation in rat liver polysomes in a manner analogous to dimethylnitrosamine. These acute structural and biochemical

FIG. 10.—Ribosomal patterns from post mitochondrial supernatant fractions of livers from rats killed 6 h following treatment with (a) saline; (b) 1-methylhydrazine; (c) 1,1-dimethylhydrazine; (d) 1,2-dimethylhydrazine.

changes found in the liver are consistent with the severe hepatic damage reported to follow repeated injections of 1,2-dimethylhydrazine in mice by Haase *et al.* (1973). It is suggested that alkylation of tissue components, including DNA, is a major factor in carcinogenesis by 1,2-dimethylhydrazine.

The authors wish to thank Mr A. Barron for the photo and electron micrographs. A. H. holds the Countess of Lisburn Memorial Studentship. This research was generously supported by the Cancer Research Campaign of Great Britain.

REFERENCES

ARGUS, M. F. & HOCH-LIGETI, C. (1961) Comparative Study of the Carcinogenic Activity of Nitrosamines. *J. natn. Cancer Inst.*, **27**, 695.

BANASCH, P. & REISS, W. (1971) Histogenese und Cytogenese Cholangiocellulärer Tumoren bei Nitrosomorpholin-vergifteten Ratten. Zugleich ein Beitrag zur Morphogenese der Cystenleber. *Z. Krebsforsch.*, **76**, 193.

BARNES, J. M. & MAGEE, P. N. (1954) Some Toxic Properties of Dimethylnitrosamine. *Br. J. ind. Med.*, **11**, 167.

BRAY, G. A. (1960) A Simple Efficient Liquid Scintillator for Counting Aqueous Solutions in a Liquid Scintillation Counter. *Anal. Biochem.*, **1**, 279.

CRADDOCK, V. M. (1968) The Effect of *N'*-Nitro-*N*-Nitroso-*N*-methylguanidine on the Liver after administration to the Rat. *Experientia*, **24**, 1148.

DOST, F. N., REED, D. J. & WANG, C. H. (1966) The Metabolic Fate of Monomethylhydrazine and Unsymmetrical Dimethylhydrazine. *Biochem. Pharmac.*, **15**, 1325.

DRUCKREY, H. (1970) Production of Colonic Carcinomas by 1,2-Dialkylhydrazines and Azoalkanes. In *Carcinoma of the Colon and Antecedent Epithelium*. Ed. W. J. Burdette. Springfield, Ill: Thomas, p. 267.

DRUCKREY, H., PREUSSMANN, R., SCHMÄHL, D. & MULLER, M. (1961) Chemische Konstitution und Carcinogene Wirkung bei Nitrosaminen. *Naturwissenschaften*, **48**, 134.

DRUCKREY, H., PREUSSMANN, R., MATZKIES, F. & IVANKOVIC, S. (1967) Selektive Erzeugung von Darmkrebs bei Ratten durch 1,2-Dimethylhydrazin. *Naturwissenschaften*, **54**, 285.

EMMELOT, P. & BENEDETTI, E. L. (1960) Changes in the Fine Structure of Rat Liver Cells Brought about by Dimethylnitrosamine. *J. biophys. biochem. Cytol.*, **7**, 393.

GANOTE, E. & ROSENTHAL, A. S. (1968) Characteristic Lesions of Methylazoxymethanol Induced Liver Damage. A Comparative Ultrastructural Study with Dimethylnitrosamine, Hydrazine Sulphate and Carbon Tetrachloride. *Lab. Invest.*, **19**, 382.

GORNALL, A. G., BARDAWILL, C. S. & DAVID, M. M. (1949) Determination of Serum Proteins by Means of the Biuret Reaction. *J. Biol. Chem.*, **177**, 751.

HAASE, P., COWEN, D. M., KNOWLES, J. C. & COOPER, E. H. (1973) Evaluation of Dimethylhydrazine induced Tumours in Mice as a Model System for Colorectal Cancer. *Br. J. Cancer*, **28**, 530.

HAWKS, A. & MAGEE, P. N. (1974) The Alkylation of Nucleic Acids of Rat and Mouse *in vivo* by the Carcinogen 1,2-Dimethylhydrazine. *Br. J. Cancer*, **30**, 440.

HAWKS, A., SWANN, P. F. & MAGEE, P. N. (1971) Probable Methylation of Nucleic Acids of Mouse Colon by 1,2-Dimethylhydrazine *in vivo*. *Biochem. Pharmac.*, **21**, 432.

JEFFERSON, L. S., WOLPERT, E. B., GIGER, K. E. & MORGAN, H. E. (1971) Regulation of Protein Synthesis in Heart Muscle. III. Effect of Anoxia on Protein Synthesis. *J. Biol. Chem.*, **246**, 2171.

KELLY, M. G. & O'GARA, R. W. (1965) Carcinogenic Activity of N-Isopropyl-(2-methylhydrazine)-p-toluamide HCl (MIH RO 4-6467, NSC 77 213). *Proc. Am. Ass. Cancer Res.*, **6**, 34.

KELLY, M. G., O'GARA, R. W., YANCEY, S. T., GADEKAR, K., BOTKIN, C. & OLIVERO, V. T. (1969) Comparative Carcinogenicity of *N*-Isopropyl-α-(2-methylhydrazine)-p-toluamide HCl (Procarbazine Hydrochloride), its Degradation Products, Other Hydrazines and Isonicotinic Acid Hydrazide. *J. natn. Cancer Inst.*, **42**, 337.

KRÜGER, F. W., WEISSLER, M. & RÜCKER, W. (1970) Investigation of the Alkylating Action of 1,1-dimethylhydrazine. *Biochem. Pharmac.*, **19**, 1825.

LAQUEUR, G. L. (1965) The Induction of Intestinal Neoplasms in Rats with the Glycoside Cycasin and its Aglycone. *Virchows Arch. path. Anat.*, **340**, 151.

LAQUEUR, G. L., MICKELSON, O., WHITING, M. G. & KURLAND, L. T. (1963) Carcinogenic Properties of Nuts from *Cycas circinalis* L. Indigenous to Guam. *J. natn. Cancer Inst.*, **31**, 919.

LEAVER, D. D., SWANN, P. F. & MAGEE, P. N. (1969) The Induction of Tumours in the Rat by a Single Oral Dose of N-Nitrosomethylurea. *Br. J. Cancer*, **23**, 117.

LÖHRS, U., WIEBECKE, B. & EDER, M. (1969) Morphologische und autoradiographische Untersuchung der Darmschleimhaut veranderungen nach einmaliger injektion von 1,2-Dimethylhydrazin. *Z. ges. exp. Med.*, **151**, 297.

LUFT, J. H. (1961) Improvements in Epoxy Embedding Methods. *J. biophys. biochem. Cytol.*, **9**, 409.

MAGEE, P. N. (1958) Toxic Liver Injury. Inhibition of Protein Synthesis in Rat Liver by Dimethylnitrosamine *in vivo*. *Biochem. J.*, **70**, 606.

MAGEE, P. N. & BARNES, J. M. (1956) The Production of Malignant Primary Hepatic Tumours in the Rat by Feeding Dimethylnitrosamine. *Br. J. Cancer*, **10**, 114.

MAGEE, P. N. & BARNES, J. M. (1962) Induction of Kidney Tumours in the Rat with Dimethylnitrosamine (*N*-nitrosodimethylamine). *J. Path. Bact.*, **84**, 19.

MAGEE, P. N. & BARNES, J. M. (1967) Carcinogenic Nitroso Compounds. *Adv. Cancer Res.*, **10**, 163.

MCLEAN, E., BRAS, G. & MCLEAN, A. E. M. (1965) Veno-occlusive Lesions in Rats after 30 mg/kg Dimethylnitrosamine. *Br. J. exp. Path.*, **46**, 367.

MILLONIG, G. (1961) Advantages of a Phosphate buffer for $OsO_4$ Solutions in Fixation. *J. appl. Physics*, **32**, 1637.

MIRVISH, S. S., CHEN, L., HARAN-GHERA, N. & BERENBLUM, I. (1969) Comparative Study of Lung Carcinogenesis, Promoting Action in Leukemogenesis and Initiating Action in Skin Tumorigenesis by Urethane, Hydrazine and Related Compounds. *Int. J. Cancer*, **4**, 318.

MUKERJEE, T., GUSTAFFSON, R. G., AFZELIUS, B. A. & ARRHENIUS, E. (1963) Effects of Carcinogenic Amines on Amino Acid Incorporation by Liver Systems. II. A Morphological and Biochemical Study on the Effect of Dimethylnitrosamine. *Cancer Res.*, **23**, 944.

OSSWALD, H. & KRÜGER, F. W. (1969) Cancerogene Wirkung von 1,2-Dimethylhydrazin beim Goldhamster. *Arzneimittelforsch.*, **19**, 1891.

PEGG, A. E. & HAWKS, A. (1971) Increased Transfer Ribonucleic Acid Methylase Activity in Tumours Induced in the Mouse Colon by the Administration of 1,2-Dimethylhydrazine. *Biochem. J.*, **122**, 121.

ROE, F. J. C., GRANT, G. A. & MILLICAN, D. M. (1967) Carcinogenicity of Hydrazine and 1,1-Dimethylhydrazine for Mouse Lung. *Nature, Lond.*, **216**, 375.

SHANK, R. C. & MAGEE, P. N. (1967) Similarities between the Biochemical Activity of Cycasin and Dimethylnitrosamine. *Biochem. J.*, **105**, 521.

SPJUT, H. J. & NOALL, M. R. (1971) Experimental Induction of Tumors of the Large Bowel of Rats. A Review of the Experience with 3′-2′-dimethyl-4-aminobiphenyl. *Cancer, N.Y.*, **28**, 29.

SWANN, P. F. & MAGEE, P. N. (1969) Induction of Rat Kidney Tumours by Ethyl Methanesulphonate and Nervous Tissue Tumours by Methyl Methanesulphonate and Ethane Methylsulphonate. *Nature, Lond.*, **223**, 947.

SVOBODA, D. & HIGGINSON, J. (1968) A Comparison of Ultrastructural Changes in Rat Liver due to Chemical Carcinogens. *Cancer Res.*, **28**, 1703.

SVOBODA, D., RACELA, A. & HIGGINSON, J. (1967) Variations in Ultrastructural Nuclear Changes in Hepatocarcinogenesis. *Biochem. Pharmac.*, **16**, 51.

TOTH, B. (1972) Hydrazine, Methylhydrazine and Methylhydrazine Sulfate Carcinogenesis in Swiss

Mice. Failure of Ammonium Hydroxide to Interfere in the Development of Tumors. *Int. J. Cancer*, **9**, 109.

TOTH, B. (1973) 1,1-Dimethylhydrazine (Unsymmetrical) Carcinogenesis in Mice. Light Microscope and Ultrastructural Studies on Neoplastic Blood Vessels. *J. natn. Cancer Inst.*, **50**, 187.

TOTH, B. & SHIMIZU, H. (1973) Methylhydrazine Tumorigenesis in Syrian Golden Hamsters and the Morphology of Malignant Histiocytomas. *Cancer Res.*, **33**, 2744.

VILLA-TREVINO, S. (1967) Possible Mechanisms of Inhibition of Protein Synthesis by Dimethylnitrosamine. *Biochem. J.*, **105**, 625.

WEIL, C. S. (1952) Tables for Convenient Calculation of Median-effective Dose ($LD_{501}$ or $ED_{50}$) and instructions in their Use. *Biometrics*, **8**, 249.

WIEBECKE, B., LÖHRS, V., GIMMY, J. & EDER, M. (1969) Erzeugung von Darmtumoren bei Mausen durch 1,2-Dimethylhydrazin. *Z. ges. exp. Med.*, **149**, 277.

ZEDECK, M. S., STERNBERG, S. S., POYNTER, R. W. & MCGOWAN, J. (1970) Biochemical and Pathological Effects of Methylazoxymethanol Acetate, a Potent Carcinogen. *Cancer Res.*, **30**, 801.